# Paradoxical Hidradenitis Suppurativa during Biologic Therapy, an Emerging Challenge: A Systematic Review

**DOI:** 10.3390/biomedicines10020455

**Published:** 2022-02-16

**Authors:** Angelo Ruggiero, Fabrizio Martora, Vincenzo Picone, Laura Marano, Gabriella Fabbrocini, Claudio Marasca

**Affiliations:** Section of Dermatology, Department of Clinical Medicine and Surgery, University of Naples Federico II, 80131 Napoly, Italy; angeloruggiero1993@libero.it (A.R.); fabriziomartora92@libero.it (F.M.); vince.picone95@gmail.com (V.P.); lauramarano94@gmail.com (L.M.); gafabbro@unina.it (G.F.)

**Keywords:** paradoxical hidradenitis, hidradenitis suppurativa, paradoxical reaction, biologics, adalimumab, infliximab, secukinumab, ixekizumab, etanercept, anti TNF, anti IL 17

## Abstract

Hidradenitis suppurativa (HS) is a chronic, inflammatory skin disease usually occurring after puberty with painful, deep-seated, inflammatory lesions in the apocrine gland-bearing areas of the body. Although HS pathogenesis is still unproven, recent major research advantages have increased our knowledge of the mechanisms behind HS lesions. Particularly, follicular occlusion followed by follicular rupture has been shown to be crucial to HS development, leading to immune response activation, and resulting in typical clinical HS lesions. Moreover, an increased and imbalanced cytokine production, such as interleukin (IL) 17 and tumor necrosis factor (TNF) α, may play a role in HS. In recent years, paradoxical adverse events have been described during treatment. Since the recent increased use of biologic treatments in HS, an increased number of paradoxical HS occurrences have been reported. In this review, we analyzed all current data on paradoxical HS triggered by biological drugs.

## 1. Introduction

Hidradenitis suppurativa (HS), also known as acne inversa, is a chronic, inflammatory skin disease usually occurring after puberty with painful, deep-seated, inflammatory lesions in the apocrine gland-bearing areas of the body [1,2]. HS affects about 1% of the global population. HS typically occurs after puberty, with the average age of onset in the second or third decades of life, and with a female predominance [2]. Although HS pathogenesis is still unproven, recent major research advantages have increased our knowledge of the mechanisms behind HS lesions. Particularly, follicular occlusion followed by follicular rupture has been shown to be crucial to HS development, leading to immune response activation, and resulting in typical clinical HS lesions [2,3,4]. Moreover, an increased and imbalanced cytokine production—such as interleukin (IL) 17 and tumor necrosis factor (TNF) α—as well as an unopposed type I interferon production, and a change toward a helper T cell 1/helper T cell 2 profile, may all play a role in HS and psoriasis [4,5]. Furthermore, in the last few years, many studies have hypothesized that HS may be triggered by genetic and environmental factors, including drugs [3]. Moreover, the de novo onset of HS has been reported after the administration of biologic treatments for chronic inflammatory diseases. Indeed, patients treated with biologic therapies for chronic inflammatory disorders may experience the onset of new skin diseases as paradoxical reactions. These are defined as the occurrence, during treatment with biologics, of a disease that is usually responsive to this class of drug [6,7]. Psoriasiform reaction, HS, uveitis, inflammatory bowel diseases, and Pyoderma Gangrenosum were the most commonly described paradoxical reactions during biological treatment [8]. In particular, the most commonly reported was psoriasis, which is a paradoxical skin reaction since it may be both induced and treated with anti-TNF [6,7]. In recent years, even more paradoxical adverse events have been described during treatment [7,8,9]. Since the recent increased use of biologic treatments in HS, an increased number of paradoxical HS occurrences have been reported. To date, TNF inhibitors represent the only class of biologics used in HS, particularly, adalimumab is the only biologic agent formally approved for the management of moderate to severe HS [10,11,12]. Although the use IL 17 inhibitors in HS is still off-label, in the literature, data are growing regarding their efficacy (particularly for secukinumab and ixekizumab), linked with an increasing number of paradoxical HS reactions [5,10,11]. In this study, we carried out a systematic review of the literature evaluating the reported cases of paradoxical HS developed during biologic therapies.

## 2. Materials and Methods

A systematic review of the scientific literature of case reports, case series, epidemiological studies, reviews and systematic reviews regarding paradoxical HS with biologic drugs was performed.

Studies were identified, screened and extracted for relevant data following the PRISMA (Preferred Reporting Items for Systematic Reviews and Meta-Analyses) guidelines.

**Figure 1:**
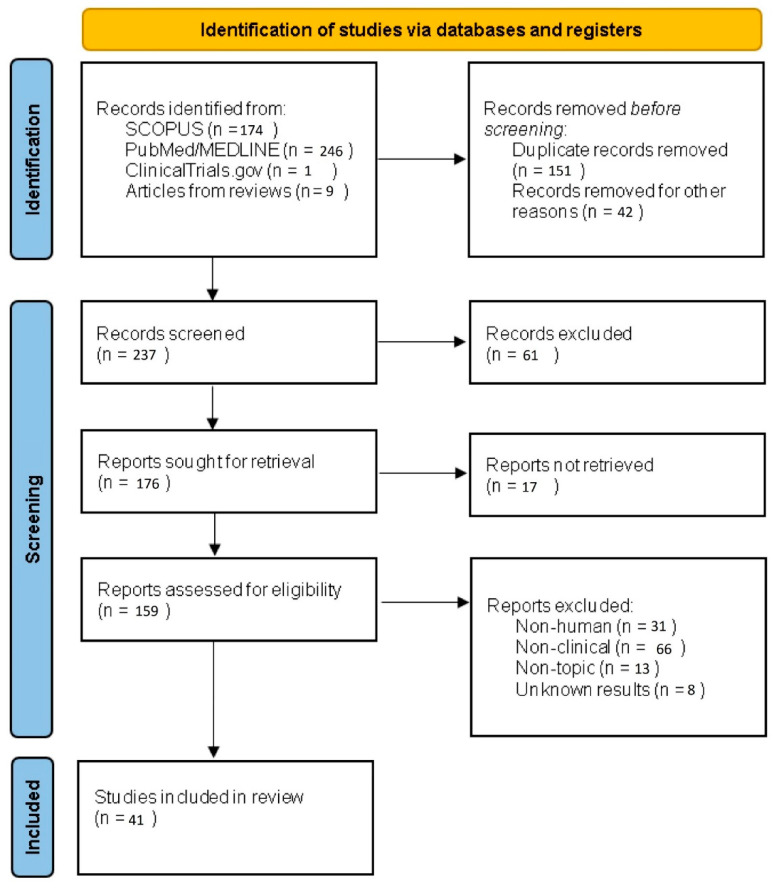
PRISMA flow diagram depicting the process of the literature search.

Flow chart of the systematic literature search according to PRISMA (Figure 1).

The study was registered in PROSPERO: the registration number is 299668.

In accordance with the 2020 edition of Preferred Reporting Items for Systematic Reviews and Meta-Analyses guidelines, a systematic search was performed using PubMed, Scopus, and Cochrane Library from their inception to 30 July 2021, using Medical Subject Headings (MeSH) terms (if applicable) and medical terms for the concepts of paradoxical HS.

Three authors (AR, CM and FM) independently screened the identified titles and abstracts for potentially eligible studies. The same two authors subsequently evaluated full-text articles reporting potentially eligible studies to make final decisions about inclusion. Any conflict of judgment was discussed and resolved between the two primary reviewers; when inconclusive, a fourth reviewer’s judgment (GF) was decisive. The following data were extracted from the included studies by the first and the second reviewer (AR and CM) and cross-checked by the third reviewer (FM): study characteristics, study group characteristics, reference test features, index test features, and numbers needed for reconstructing table (AR, FM, VP, GF, LM, and CM).

Regarding the review of the literature, a search of the Pubmed, Embase and Cochrane Skin databases (until July 2021) was performed, using the following research terms: “hidradenitis suppurativa”, “acne inversa”, “apocrine acne”, “apocrinitis”, “hidradenitis axillaries”, “Verneuil’s disease”, “adalimumab”, “anti TNF”, “rituximab”, “secukinumab”, “ixekizumab”, “ustekinumab”, “etanercept”, “brodalumab”, “guselkumab”, “risankizumab”, “certolizumab”, “JAK inhibitors”, “tildrakizumab”, “anti IL 17”, “anti IL 12 23”, “anti IL 23” and “paradoxical reactions or drug induced”.

Search criteria were the following:

(a) Articles published in scientific journals included in MEDLINE or EMBASE databases;
(b) Articles written in English: all types of epidemiological studies were included.

Title and abstract review were performed. Therapies that could be categorized as traditional Chinese medicine, herbal medicine or Ayurveda/Ayurvedic medicine were excluded. Articles regarding non-new onset HS during biologic treatments were excluded. The article was based on previously conducted studies.

For each patient, the following data were collected: age, sex, associated inflammatory disorder, hidradenitis risk factors, type of treatment and duration, Hurley stage, switch/discontinuation/maintenance of the treatment and response. All collected data are reported in Table 1.

## 3. Results

A total of 41 cases of paradoxical HS cases in patients treated with biological therapy, from both reports and studies, were included in the review. For all of the considered cases data about HS outcomes were available. Among patients developing paradoxical HS, the median age was 37.5 ± 63 years, with a predominance of female gender (79%, n = 32). Interestingly, Crohn’s disease (CD) was reported as a comorbidity in 51% (n = 21). Other relevant biologic-treated comorbidities were rheumatoid arthritis and assail spondylitis, both reported in 12.2% of cases (n = 5). Adalimumab was the most common involved biologic agent (56%; n = 23), followed by infliximab (21.9%, n = 9). Mean duration of biological treatment prior to the manifestation of paradoxical HS was 24 months (1–72 months). Smoking habit was reported in 21 cases (51%), and overweight or obesity in 18 cases (43.9%), which were the most common documented HS risk factors. The distribution according to Hurley severity stage was: stage I = 36.6% (n = 15); II = 54% (n = 22); and III = 9.4% (n = 4) The involved biological drug was maintained in 21 of 41 patients (51.2%), obtaining partial responses in 7 of them (33.3%) and complete remission in 3 (14.3%). In the remaining 20 patients the biological agent was stopped or switched; in 6 of them the biological agent was suspended (30%), achieving improvement with partial remission in 2/6 (33.3%), and with complete remission in 4/6 (66.7%). In the other 14 patients, the biological drug was switched to another biological agent; the most used was ustekinumab (57.1%) n = 8, with all cases reaching clinical improvement (except one of the cases where evolution was still pending) and 7/8 (87.5%) achieving partial or complete remission. In one other case, the drug was switched to abatacept showing complete remission. In another case it was switched to etanercept showing complete remission, and in another case it was switched to azathioprine showing complete remission. Adalimumab was reintroduced in three patients with controlled disease, leading to HS relapse in all of them.

All the reported cases of paradoxical HS improved after the discontinuation of the involved biological drug.

Characteristics of the cases are detailed in Table 1.

### 3.1. Anti-TNF α

TNF α is considered as a primary driver of the inflammatory process behind HS [12]. Indeed, significantly higher TNF α concentration was found in lesional HS skin, perilesional HS skin and the serum of HS patients compared with healthy controls, although no significant correlation between TNF α levels and disease severity, including the intensity of inflammation, has been demonstrated [21,22]. Moreover, the blockage of TNF α showed significant improvements on HS. Hence, anti-TNF α represents the only approved biologic for moderate and severe forms of HS. Many cases of paradoxical skin reactions—especially psoriasis—secondary to the use of TNF α inhibitors have already been reported, as well as other immune-mediated inflammatory diseases. Moreover, anti-TNF also represents the most reported biologic class linked with paradoxical HS.

### 3.2. Adalimumab

Adalimumab, a fully human monoclonal IgG1 antibody directed toward membrane-bound TNF α is the only FDA-approved biologic therapy for moderate to severe HS [6,23,24]. The approved standard dosing regimen of adalimumab is 160 mg at baseline (week 0), and 80 mg at week 2, followed by 40 mg weekly from week 4 [16,25,26,27,28]. Some cases of paradoxical HS have been reported in patients suffering from chronic inflammatory bowel diseases treated with adalimumab who developed moderate to severe forms of paradoxical HS [20,29]. Indeed, in a retrospective study including 25 patients affected by autoimmune diseases, Faivre et al. reported 12 cases of paradoxical HS during adalimumab treatment, which showed improvements after adalimumab suspension and a HS relapse when restarting the treatment [6]. Furthermore, Delobeau et al. reported four cases of patients suffering from autoimmune diseases (such as Crohn’s Disease, juvenile idiopathic arthritis, severe plaque psoriasis and ankylosing spondylitis) developing paradoxical HS under adalimumab [13]. In all these studies, females were the dominant gender [6,13,14,15,20,29]. Regarding the need for adalimumab discontinuation or not, the recently proposed algorithm management of paradoxical HS proposed to continue adalimumab in mild forms and discontinue treatments only in the case of progressive worsening or in more severe forms. In all cases, additional treatment was required to improve HS outcomes.

### 3.3. Etanercept

Etanercept is a dimeric fusion protein that binds to the soluble and leukocyte membrane-bound TNF α receptor. FDA approved the use of etanercept to treat moderate to severe rheumatoid arthritis, moderate to severe polyarticular juvenile rheumatoid arthritis, psoriatic arthritis, ankylosing spondylitis, and psoriasis. To date, fewer cases of paradoxical HS have been reported with etanercept than adalimumab [16,30]. Particularly, Pellegrino M. et al., reported the case of a 30-year-old male diagnosed with juvenile idiopathic arthritis who developed paradoxical HS lesions during etanercept treatment [30]. Only three other cases have been reported of paradoxical HS during etanercept in patients suffering from other autoimmune diseases [16]. In all cases, etanercept suspension lead to an improvement of HS lesions and a remission after the addition of other treatments [16,30].

### 3.4. Infliximab

Infliximab is a chimeric monoclonal antibody approved to treat a number of autoimmune and chronic inflammatory diseases, including CD, ulcerative colitis, rheumatoid arthritis, ankylosing spondylitis, psoriasis, psoriatic arthritis, and Behçet’s disease. Even if adalimumab still represents the only FDA-approved biologic for HS, infliximab has been demonstrated to be an effective treatment for HS and, as confirmed from recent guidelines, it could be proposed as a second line biologic treatment for moderate to severe HS (recommendation grade B; evidence level: 2) [31]. Although lower than for adalimumab, some cases of paradoxical HS have been reported [31,32]. Particularly, Savaşan et al. [31] reported the case of a young patient suffering from CD who developed, for the first time, HS lesions as a paradoxical effect of infliximab treatment [33]. Similarly, cases of paradoxical HS were reported in patients suffering from CD treated with infliximab [32,33,34,35,36].

### 3.5. Other Biologic Classes

Although anti-TNF still represents the only FDA-approved class of biologic treatment for moderate to severe HS, new emergent treatments have been shown to be potentially effective therapies in the management of unresponsive HS forms [10,11,17]. Hence, with the progressively increased use of these new drugs, the cases of paradoxical HS reactions are slowly increasing in the literature.

### 3.6. Anti-IL-17

Increased IL 17 levels have recently been found in serum of HS patients linked with a significantly increased number of IL 17-producing cells in both affected and non-affected skin of HS patients [3]. Indeed, recent clinical trials with secukinumab have been performed to evaluate its efficacy and safety in moderate to severe HS. Although the use of anti-IL 17 is still off-label in HS, data are growing in the literature regarding their efficacy (particularly for secukinumab, a fully human IgG1 kappa monoclonal antibody targeting IL 17A) [5,10,11,17,37]. With regard to paradoxical HS, few cases have been reported with the use of secukinumab [13,17,18,37]. Particularly, Navarro et al. reported the case of a patient suffering from severe psoriasis unresponsive to anti-TNF, who developed paradoxical HS lesions after secukinumab treatment [17]. Moreover, in a previously published article (Marasca et al., 2019 [38]) we highlighted the immunological complexity behind HS, showing the potential double pathophysiological face of secukinumab in HS, describing a case of secukinumab-induced HS and a case of HS provoked by adalimumab treatment and controlled with secukinumab therapy [18]. Hence, the real role of secukinumab in the management of HS is still not well clarified, and it is a possible trigger for paradoxical HS lesions.

### 3.7. Anti-IL-12/23 and Anti-IL-23

Ustekinumab is a fully human monoclonal antibody against IL 12/23, acting on Th1 and Th17 pathways [39]. Although the use of anti-IL 12/23 in the management of moderate to severe HS is still off-label, several case series and studies have been reported about its possible efficacy in unresponsive and severe HS [40,41]. Indeed, in the literature, some case reports showed the possible role of ustekinumab in HS management [41,42]. However, a phase II open label study evaluating ustekinumab use in moderate to severe HS showed only limited benefits [42]. With regard to paradoxical HS, to date only one case has been described. Particularly, Gkini et al. reported the case of a 19-year-old female treated with ustekinumab for severe psoriasis, who developed HS lesions (Hurley stage II), requiring a surgical approach [19]. Anti-IL 23 drugs (guselkumab, risankizumab, tildrakizumab) represent the latest class of biologics approved for the treatment of moderate to severe psoriasis [43,44,45,46]. To date, the efficacy and safety of anti-IL 23 in the management of HS have been described only in some case series, showing contrasting results [47,48,49,50]. Indeed, the precise role of IL 23 in HS is not yet fully understood. However, previous findings suggest that the IL 23/Th17 pathways seem to be crucial in the pathogenesis of HS, including IL 23, suggesting a potential role of anti-IL 23 in HS management [50]. With regard to paradoxical HS during anti-IL 23 treatment, no cases have been reported to date.

## 4. Discussion

HS, also known as acne inversa, is a chronic inflammatory skin disease affecting about 1% of the global population [1,2]. HS typically occurs after puberty, with the average age of onset in the second or third decades of life, and with a female predominance [2]. Although the pathogenesis of HS is not completely understood, recent research advantages have led to a greater insight into the mechanisms behind the disease [3]. Particularly, the primary defect in HS may be represented by hair follicle occlusion, followed by follicular rupture, and an abnormal immune response. These alterations are necessary conditions for the development of clinical HS lesions [2]. Many factors have been found to act as triggers for HS, including both environmental factors (such as microbial colonization, cigarette smoking, and obesity) and a specific genetic signature. All these factors may start an aberrant immune response, resulting in altered cytokines production. Indeed, although the exact mechanism through which cytokines and immune pathways drive inflammation in HS continue to be investigated, different pathways have been found to be crucial in the development of HS. Particularly, TNF-α, a proinflammatory cytokine produced by innate and adaptive immune cells, has been shown to play a central role in HS [2,3,4]. These findings result in the approval of targeted therapy (anti-TNF) for the management of most severe forms of HS [3].

Paradoxical reactions during biologic treatments may be defined as the appearance or exacerbation of a pathological condition that usually responds to this class of drug while treating a patient for another condition [38].

Typical examples of paradoxical adverse effect are: psoriasis (such as palmoplantar pustular), psoriasiform reactions, and HS. These reactions have been mainly reported in patients during biologic treatment for rheumatoid arthritis or inflammatory bowel disease [29]. However, the exact underlying physiopathologal mechanism behind these paradoxical reactions is unknown, although it has been showed that multiple immunological pathways may be involved. Particularly, one of the most important factors behind paradoxical reactions could be cytokine imbalance, which could play a key role in the development of these paradoxical reactions [6].

Although the pathogenesis of paradoxical HS induced by biologics is still unproven, it has been observed that, in genetically predisposed individuals, the introduction of biologics may induce a modulation of the innate immune system, leading to an alteration of the cytokine balance, and an increase in pro-inflammatory cytokines [38]. Nowadays, adalimumab represents the most commonly used biologic treatment involved in the onset of paradoxical HS. Particularly, a recently published multicenter retrospective study reported paradoxical HS under biological agents, with adalimumab being responsible for 48% of new HS onset cases [6]. TNF α inhibitors may cause modification of the local cytokine balance and activate alternate pathways, namely type 1 interferon and the IL 1-related family of inflammation mediators, in selected patients. The production of IL 1b is increased up to 54-fold in conventional HS skin, compared with healthy control skin, and IL 1 receptor antagonists have shown efficacy in HS. Individual susceptibility is required if further up-regulation of the IL 1 pathway is involved in TNF α inhibition, because adalimumab reduces IL 1b levels in conventional HS. It is also important to consider that, due to the larger use of this class in the management of HS, the paradoxical reactions after their use (particularly with adalimumab) are reported most frequently, without implying that there is a higher individual risk of causing paradoxical HS. As consequence, a challenging issue is whether or not to continue anti-TNF treatment after the onset of paradoxical HS. Indeed, a management algorithm for paradoxical HS has recently been proposed which suggested to continue anti-TNF treatment in mild paradoxical HS forms, suggesting a suspension in the case of a progressive worsening, or at the beginning in more severe forms [6]. To date, cases of paradoxical HS have been reported with the use of adalimumab, etanercept and infliximab.

In order to clarify the role of adalimumab and other anti-TNF involved in HS paradoxical reactions, including infliximab and etanercept, an imbalance has been supposed, induced by TNF blockage in the cytokine pool and a consequent activation of type I interferon or IL 1 beta, together with the possible role of occult infections [51,52]. Although adalimumab still represents the only FDA-approved biologic therapy in HS management, it has also been recently proposed that a key role is played by the IL 17 pathway in HS pathogenesis. Indeed, increased serum levels of IL 17 have been found in patients with HS, as well as a significantly increased quantities of IL 17-producing cells in both lesional and perilesional HS skin, compared with healthy subjects [2,3]. Indeed, anti-IL 17s, particularly secukinumab and ixekizumab, have been proposed as treatment options for moderate to severe HS, being also reported as possible effective therapies in some case reports [4,5,6,7]. Furthermore, secukinumab is being investigated in a phase III trial for HS treatment. Although only a few cases have been reported, anti-IL 17 has been linked to the development of paradoxical HS [5,10,11,17,18,37,38]. However, as for anti-TNF, the hypotheses to explain paradoxical HS are still scant. Interestingly, among patients experiencing paradoxical HS reactions, we found that CD was the most frequent comorbidity (48.8%, n = 21), followed by rheumatoid arthritis and assail spondylitis, both reported in 11.6% of cases (n = 5). Indeed, HS and inflammatory bowel diseases, such as CD, share genetic susceptibility, common clinical manifestations at the gut and skin, and some immunologic features. Some genes, for example SULT1B1 (OMIM 608436) and SULT1E1 (OMIM 600043), have been associated with HS and inflammatory bowel disease. Emerging studies have shown that HS and inflammatory bowel disease are both diseases characterized by immune dysregulation. Cytokine abnormalities, such as the elevation of IL 1, IL 6, IL 17, IL 23 and the tumor necrosis factor, are involved in HS and inflammatory bowel disease. Alteration of the microbiota, resulting in immune dysregulation, also appears to play an important role in both diseases [53]. However, the exact mechanism linking paradoxical HS manifestation and CD is still unclear. With regard to the treatment of paradoxical HS reactions, Faivre et al. [6] have recently proposed a management algorithm, suggesting that the involved biologic agent may be continued in mild HS forms, while the discontinuation should be recommended in more severe forms or in cases of progressive worsening [6]. This management algorithm may be applied to all biologic agents involved in HS reaction, adding in all cases a specific HS treatment. We can highlight from our review that, regarding the therapeutic switch, the use of USK has reported good results in patients who were previously treated with ADA. This finding is very important for the literature because it could open new therapeutic scenarios in HS. Certainly, further studies are needed to confirm this finding.

## 5. Limitations of the Study

The main limitation of our study is certainly represented by the limited number of cases we considered, given the retrospective nature of our study, and the few data currently present in the literature on the topic. In addition, we could not directly intervene with patients in treatment decisions. Another limitation may be represented by the fact that these patients had various comorbidities, including common risk factors for chronic inflammatory diseases such as smoking and obesity, and this may represent a selection bias. In addition, because HS is a chronic inflammatory disease, it is often associated with other inflammatory conditions.

## 6. Conclusions

Our study mainly showed that ADA is the biological drug most frequently involved in paradoxical reactions. Certainly, the results of the ADA data were influenced by the high number of patients who had undergone therapy with ADA taken in our data collection; however, this data is still interesting because, at the same time, ADA is the only biological drug currently available for HS. Consequently, our review aims to encourage new studies on this topic in order to better understand these paradoxical reactions, resulting in a faster diagnosis and an earlier treatment for this common condition.

## 7. Future Directions

HS represents a chronic skin condition that has a significant impact on patients’ quality of life, being one of the least responsive dermatologic diseases to currently available therapies. For this reason, it is important to prevent any recurrence of this disease whenever possible. Patients with chronic inflammatory skin conditions treated with biotechnology drugs are at risk of developing paradoxical HS, as highlighted in our review. Therefore, we believe it is important for the future to investigate aspects such as predictive factors for the development of these reactions, to modify them if possible or, if not, to choose the right therapeutic approach for the patient. Thus, establishing a specific management algorithm would be helpful in managing the onset of paradoxical HS. Faster recognition of these paradoxical reactions by dermatologists would allow for faster correct treatment and better clinical outcomes.

## Figures and Tables

**Table 1:** Reported cases of paradoxical hidradenitis suppurativa in patients treated with biologic agents.

Refs.	Sex/Age	Associated Inflammatory Disorder	Hidradenitis Risk Factors	Treatment Duration (Months)	Hurley Stage	Switch/Discontinuation	Response
[6]	M/43	RA	Smoker, obese	ADA (2)	I	Switch to abatacept	CR
[6]	F/35	RA	Smoker, diabetes	IFX (9)	II	Maintained	Worsening
[6]	F/54	RA	FHS, smoker, obese, severe acne	ETN (2)	I	Maintained	Worsening
[6]	F/27	RA	Overweight, pilonidal sinus	IFX (11)	I	Maintained	Worsening
[6,13]	F/17	CJA		ADA (48)	II	Switch to ETN	CR
[6]	M/33	AS	Smoker, pilonidal sinus, obese	ADA (24)	II	Stop	PR
[11]	M/20	AS	Severe acne, dissecting cellulitis, overweight	ETN (10)	II	Maintained	SD
[6,8,13]	F/21	AS		ADA (18)	II	Switch to Ustekinumab	CR
[6]	F/24	AS	Smoker	ADA (57)	II	Maintained	PR
[6]	F/46	AS	Smoker, Obese	ADA (18)	II	Maintained	Worsening
[6]	F/28	Psoriatic arthritis	Smoker, overweight	ADA (28)	I	Maintained	SD
[6]	F/55	SAPHO	Smoker, obese	ETN (12)	I	Stop	CR
[6]	F/35	CD	Smoker, overweight	ADA (6)	I	Stop	PR
[6]	F/28	CD	Obese	ADA (10)	I	Maintained	PR
[6]	F/51	CD	Smoker, diabetes, obese	ADA (12)	III	Maintained	PR
[6]	F/29	CD	Smoker	IFX (72)	II	Switch to ADA	SD
[6]	F/23	CD	Smoker	ADA (1)	II	Maintained	PR
[6]	F/28	CD	FHS	ADA (3)	II	Maintained	PR
[6]	F/22	CD		IFX (5)	I	Maintained	Worsening
[6]	M/26	CD		IFX (42)	II	Stop	CR
[6]	F/50	CD		IFX (48)	I	Maintained	SD
[13]	F/29	CD	Smoker, Obese	ADA (7)	II	Maintained	PR
[14]	F/57	CD	Smoker	ADA (12)	II	Switch to AZA	CR
[14]	M/24	CD		ADA (9)	II	Maintained	Worsening
[15]	F/40	CD		ADA (21)	II	Stop	CR
[6]	F/49	Psoriasis	Obese	ADA (54)	I	Maintained	CR
[13]	F/51	Psoriasis	Smoker, Obese	ADA (12)	II	Maintained	CR
[9]	F/42	CD	Smoker, Obese	IFX (60)	I	Switch to Ustekinumab	CR
[9]	F/19	CD		IFX (24)	II	Stop	CR
[9]	F/22	CD	Smoker, pilonidal sinus, overweight, severe acne	IFX (12)	II	Maintained	SD
[9]	F/40	CD	Smoker, severe acne	ADA (72)	II	Switch to ustekinumab	ND
[9]	F/51	RA	Diabetes	ADA (69)	I	Maintained	PR
[16]	F/55	Psoriasis	Smoker	ETN (1)	I	Switch to ustekinumab	CR
[16]	M/53	CD	Smoker, obese	ADA (6)	II	Switch to ustekinumab	PR
[10]	M/58	Psoriasis and Psoriatic arthritis		SCK(4)	II	Switch to ustekinumab	CR
[17]	M/46	Psoriasis		SCK (32)	III	Switch to Adalimumab	CR
[18]	M/48	Psoriasis		IXZ (12)	I	Maintained	CR
[19]	F/19	Psoriasis	Smoker	USK (48)	III	Switch to Adalimumab	PR
[20]	F/38	CD		ADA (12)	III	Switch to Ustekinumab	PR
[20]	F/27	CD		ADA (40)	I	Maintained	SD
[20]	F/34	CD		ADA (48)	II	Switch to Ustekinumab	SD

DA, adalimumab; AS, ankylosing spondylitis; ATB, antibiotic therapy; AZA, azathioprine; BA, biologic agent; CD, Crohn’s disease; CJA, chronic juvenile arthritis; CR, complete remission; CS, corticosteroid therapy; ETN, etanercept; F, female; FHS, familial history of hidradenitis suppurativa; H, Hurley stage; HS, hidradenitis suppurativa; IFX, infliximab; IXZ, ixekizumab; M, male; MTX, methotrexate; ND, no data; PR, partial remission; RA, rheumatoid arthritis; Ref.: reference in which this patient is described; RTX, rituximab; SCK, secukinumab; SD, stable disease; USK, ustekinumab.
